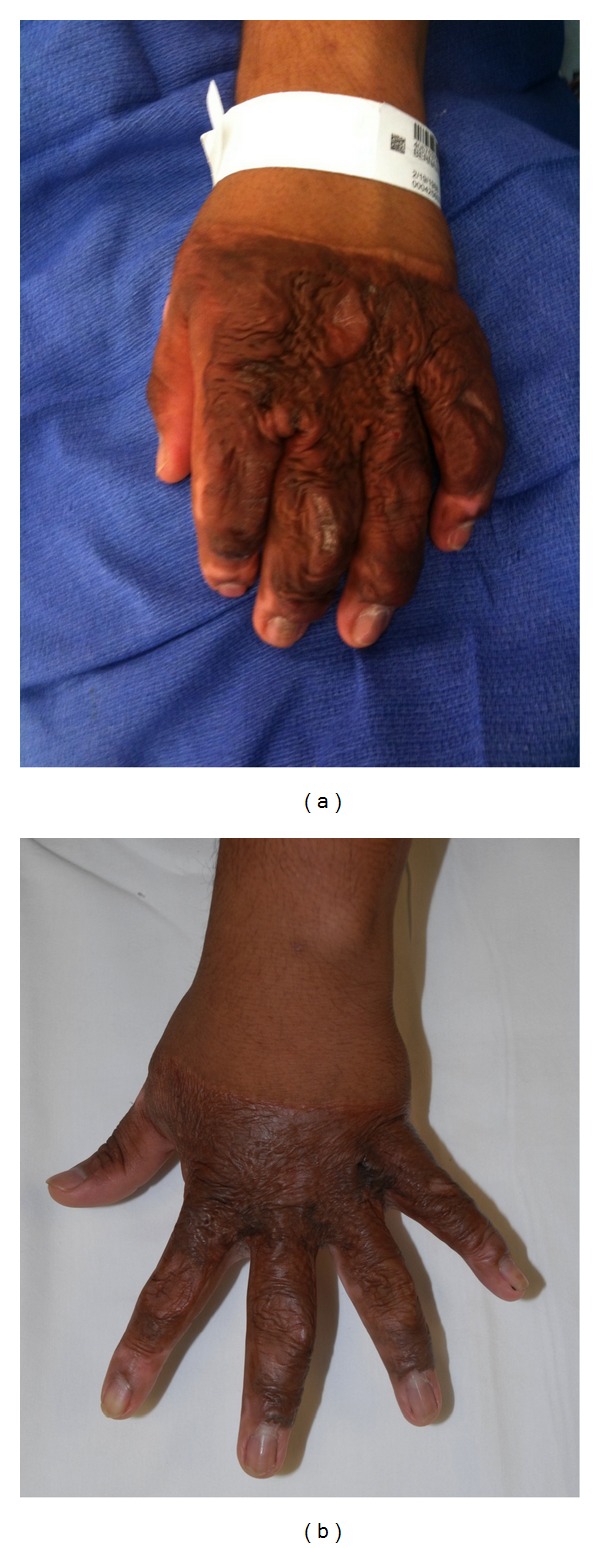# Shine on: Review of Laser- and Light-Based Therapies for the Treatment of Burn Scars

**DOI:** 10.1155/2012/243651

**Published:** 2012-06-20

**Authors:** C. Scott Hultman, Renee E. Edkins, Clara N. Lee, Catherine T. Calvert, Bruce A. Cairns

**Affiliations:** ^1^Division of Plastic Surgery, University of North Carolina School of Medicine, Chapel Hill, NC 27599-7195, USA; ^2^The NC Jaycee Burn Center, University of North Carolina, Chapel Hill, NC 27599-7195, USA

## Abstract

Restoration of form and function after burn injury remains challenging, but emerging laser and pulsed light technologies now offer hope for patients with hypertrophic scars, which may be associated with persistent hyperemia, chronic folliculitis, intense pruritis, and neuropathic pain. In addition to impairing body image, these scars may limit functional recovery, compromise activities of daily living, and prevent return to work. Three different platforms are now poised to alter our reconstructive algorithm: (1) vascular-specific pulsed dye laser (PDL) to reduce hyperemia, (2) ablative fractional CO_2_ laser to improve texture and pliability of the burn scar, and (3) intense pulsed light (IPL) to correct burn scar dyschromia and alleviate chronic folliculitis. In this paper, we will provide an overview of our work in this area, which includes a systematic review, a retrospective analysis of our preliminary experience, and interim data from our on-going, prospective, before-after cohort trial. We will demonstrate that laser- and light-based therapies can be combined with each other safely to yield superior results, often at lower cost, by reducing the need for reconstructive surgery. Modulating the burn scar, through minimally invasive modalities, may replace conventional methods of burn scar excision and yield outcomes not previously possible or conceivable.

## 1. Background

Restoration of form and function after burn injury remains challenging, but traditional and emerging laser- and light-based technologies may offer new hope for patients with burn scars.

In addition to serving as a visible reminder of the burn injury and compromising self-esteem and self-image, burn scars produce considerable functional morbidity, including contractures, hypertrophic changes, and keloid formation. Furthermore, burn scars often result in persistent hyperemia, chronic folliculitis, intense and unrelenting pruritis, and neuropathic pain. The loss of sweat glands, hair follicles, and melanocytes compromises the ability of skin, the body's largest organ, to provide thermoregulation, to resist mechanical trauma, and to protect from UV radiation. The stigmata of burn scars are plainly visible, but the injury to the patient is often more than skin deep.

Depending upon the constellation of patient symptoms and functional deficits, treatment of the burn scar involves a number of modalities [1], which may include massage and moisturizing agents, pressure garments, silicone sheeting, topical and intralesional steroids, and experimental therapies such as interferon. Surgical incision or excision of the burn scar may be necessary, and defects are reconstructed with biologic skin substitutes, split- and full-thickness skin grafts, tissue rearrangement, tissue-expanded or pedicled flaps, and even free tissue transfer. Keloids may even require perioperative radiotherapy to reduce the incidence of recurrence.

 Three different laser- and light-based technologies are now poised to dramatically alter our reconstructive algorithm and create a major paradigm shift in the management of burn scars.

- Vascular-specific pulsed dye laser (PDL) therapy to reduce hyperemia and hypertrophic scar formation.
- Ablative fractional CO_2_ laser resurfacing to help correct the abnormal texture, thickness, and stiffness of the burn scar.
- Intense pulsed light (IPL) therapy to improve burn scar dyschromia and alleviate chronic folliculitis.

**Pulsed Dye Laser Therapy**

Developed several decades ago, the vascular-specific, flashlamp-pumped 585 and 595 nm pulsed dye lasers became the standard of care in the treatment of port wine stains, capillary malformations, and some hemangiomas. This laser selectively targets hemoglobin and coagulates microvasculature in the papillary and reticular dermis, up to a depth of 1.2 mm. Hypertrophic burn scars are characterized by excessive inflammation, prolonged time to reepithelialization, overabundant collagen production, abnormal extracellular matrix remodeling, and inhibition of fibroblast apoptosis, all of which result from or are related to pathologic neovascularization. The PDL causes selective photothermolysis that induces coagulation necrosis of capillaries. When applied to burn scars, the PDL serves to extinguish this hypervascular response. Over the past 10 years, the efficacy of PDL therapy for the treatment of subacute burns and hypertrophic burn scars has been established by scores of investigators [2–8], including Donelan et al. [2], from the Shriners Burns Hospital in Boston, who present their experience with 57 patients. This group notes that PDL improves burn scar texture, pliability, erythema, pruritis, and pain, while reducing scar volume.

**Ablative/Nonablative Fractional Laser Resurfacing**

Fractional laser resurfacing, first introduced in 2005 and later refined with microfractional technology in 2007, has been largely utilized for cosmetic indications, such as treatment of photoaging, fine rhytids of the mouth and eyelids, and abnormal pigmentation. Ablative methods (specifically CO_2_ and Erbium-YAG) target intracellular water, leading to vaporization of tissue and denaturation of surrounding extracellular proteins. Nonablative lasers (1540 nm Erbium) induce coagulation only. Fractional resurfacing is theoretically attractive in the management of burn scars, because microscopic columns of abnormal dermis are vaporized or coagulated, which in turn stimulate collagen production and remodeling. Furthermore, these microscopic treatment zones (MTZs) are 70–100 micrometers in diameter and 250–800 micrometers in depth, leaving a significant amount of epidermis and dermis intact, to assist in rapid and controlled wound healing. Deeper, safe penetration with these ablative lasers may be possible by decreasing the density of the MTZs and increasing fluence. Several recent reports have demonstrated the clinical value of fractional resurfacing in hypertrophic and atrophic burn scars, with dramatic results observed even in scars that were decades old [9–12].

**Intense Pulsed Light**

Although not technically a laser, IPL delivers focused, controlled light energy through a coupling gel, across the 515–1200 nm spectrum and at a fluence of up to 40 J/cm^2^. IPL coagulates vascular lesions, removes unwanted pigment, and facilitates hair removal. Specific filters in the hand piece allow the user to select a window of wavelengths, such as 755 nm for collagen stimulation, 695 nm to remove superficial leg veins, and 515 nm to treat rosacea. The exact mechanism of action is unknown, and no negative long-term effects have been observed. Although traditionally used for cosmetic purposes, IPL may prove to be helpful in the management of burn scar dyschromia, long-standing hypervascularity, and chronic folliculitis, without the risks or downtime of PDL photothermolysis or laser resurfacing. A recent report of IPL in 109 patients with hypertrophic scars and keloids (due to traumatic wounds, burns, and surgical incisions) demonstrated improvement in 92.5% of subjects, in terms of scar height, erythema, and hardness, with a high level of patient satisfaction [13].

## 2. Systematic Review

 To determine the indications for and efficacy of laser- and light-based therapy in the treatment of hypertrophic burn scars, we first performed a systematic review to assess the quality of the literature regarding this topic [14]. Using the key words “burn,” “scar,” “hypertrophic,” “laser,” “treatment,” and “therapy,” we searched several electronic databases (PubMed, Embase, Cochrane, Google Scholar) and examined references cited in review articles. Inclusion criteria were the following: population: patients undergoing treatment of hypertrophic burn scars; intervention: laser or light-based therapies; outcomes: change in burn scar height, vascularity, stiffness, appearance, pain, and pruritis; study design: prospective or retrospective trials; language: English.

We located 329 potential articles, which were refined to 87 unique, relevant publications. After eliminating animal studies, letters, case reports, reviews, and clinical trials with an unknown or limited (<5) number of burn patients, we identified 12 articles [2, 4–6, 10, 13, 15–20] that met our inclusion criteria: 1 cohort study, 5 before-after studies, 4 controlled clinical trials, and 2 randomized controlled trials, 10 of which were prospective. All 6 of the clinical trials used internal controls, in which patients' burn scars were split into treatment *versus* no treatment areas. No publications used nontreated external controls, compared different types of laser therapies, or compared laser treatment to nonlaser treatments, such as pressure garments or silicone sheeting.

 A total of 770 patients were treated with pulsed dye laser (5 articles), erbium laser (3 articles), low-level laser therapy (2 articles), and intense pulsed light (2 articles). Assessment of outcomes was blinded in 3 articles and utilized a validated grading system in 6 articles. 11 articles demonstrated mild to moderate improvement in hypertrophic burn scars, whereas 1 article showed no sustained improvement over internal controls. The most frequent benefits were improvements in erythema, height, and pliability, noted in 5 articles, although improvements in pain, pruritis, color, and texture were also observed. Complications were reported in 18 patients, or 2.3% of the total group.

 This systematic review supports the use of laser- and light-based therapies as an efficacious and viable option for the treatment of hypertrophic burn scars. However, more robust clinical trials are needed to help determine best practices and guide clinicians regarding the timing and type of therapy.

## 3. Preliminary Experience: Retrospective Case Series

 With this information from our systematic review, we performed a retrospective analysis of our preliminary experience with laser therapy for the treatment of patients with hypertrophic burn scars. We examined the medical records of 79 burn patients who underwent 189 treatments with PDL, CO_2_, or IPL, from October 2008 through September 2010, to determine safety of combining different modalities, treatment parameters, timing of therapy, and patient satisfaction. The setting for this study was the North Carolina Jaycee Burn Center, a licensed 40-bed burn unit, with over 1000 admissions per year, verified by the American Burn Association and accredited by the American College of Surgeons. As a component of the University of North Carolina Health Care System, a public entity that reports to both the Chancellor of the University and the State Legislature, the NC Jaycee Burn Center is dedicated to providing complete care for all North Carolinians with burn injury and has extensive advocacy, prevention, research, education, and aftercare programs.

 Over the course of the two-year period, we developed treatment protocols that have remained fairly stable and extend into our current practice. All procedures are performed in the operating room with anesthesia provided by anesthesiologists or CRNAs supervised by anesthesiologists. Although topical anesthesia and oral anxiolytics might suffice in some settings, our standard practice is to use short-acting, intravenous agents such as fentanyl, versed, and propofol. For children, patients with larger burn scars, or patients with difficult airways, we utilize a laryngeal mask airway to provide a greater degree of airway security. We do not routinely use perioperative antibiotics or antiviral agents but apply bacitracin to the treated area, with resumption of occupational therapy, physical therapy, and pressure garments within several days. Patients often return to school or work the day after treatment.

 Specific lasers used include (1) the Candela 595 nm V-beam pulsed dye laser (Wayland, MA, USA), (2) the Lumenis UltraPulse fractional CO_2_ laser (Santa Clara, CA, USA), and (3) the Lumenis LUME-1 IPL/Nd:YAG/lightsheer diode Workstation (Santa Clara, CA, USA). For the PDL, fluence is selected based upon the Fitzpatrick skin type, with lower fluences used for patients with increased melanin, in an effort to reduce incidence of blistering and decrease risk of postprocedure hypo- and hyperpigmentation. Standard settings include a fluence of 6–11 J/cm^2^, 1.5 msec pulse duration, 7 mm spot size, 0–30% overlap, and 1-2 passes. End point of PDL therapy for each session is purplish discoloration of the targeted area. For the fractional CO_2_ laser, we primarily use the DeepFX*™* handpiece to treat thick, stiff scar, at the following settings: density of 15%, frequency of 600 Hz, and 12.5–17.5 mJ per micropulse. Patients with textural abnormalities may also benefit from using the ActiveFX*™* handpiece, which delivers superficial ablation, at the following settings: frequency of 150 Hz, 70–90 mJ per micropulse. Both the DeepFX*™* and ActiveFX*™* therapies can be combined, over the same area of burn scar, without increasing the risk of complications. For IPL, we typically select a filter of 515–590 nm, with a fluence of 18–24 J/cm^2^, to treat dyschromia and chronic folliculitis.

 Nearly every patient in this preliminary case series had a favorable response, with a high degree of patient and provider satisfaction. Most of the improvement in the burn scar was apparent after 1-2 sessions, but some individuals required up to 6 sessions to have a sustained and measureable response. Treatments are repeated every 4–6 weeks, until improvement plateaus. Overall, we have observed impressive improvement in pigmentation, pliability, height, vascularity, pain, and pruritis. Based upon this experience, as well as that of our prospective before-after cohort study, our current algorithm includes the following: (1) use of PDL to treat hyperemia and hypertrophic thickening, within the first 6–36 months after burn injury, (2) use of fractional ablative CO_2_ laser to treat abnormal pliability and texture, from 12 months to years after injury, and (3) use of IPL to treat burn scar dyschromia, years after injury.

## 4. Prospective, Before-After Cohort Study

 Because of the need to demonstrate quantitative changes in hypertrophic scars after therapy, we next conducted a prospective, validated, before-after cohort study of burn patients who underwent laser treatment of their burn scars [21]. From January through November 2011, we treated 142 patients with 443 lasers in 382 sessions, representing 2.7 sessions per patient. Mean age was 26.9 years, with a range of 2–69 years. The most common Fitzpatrick skin type was 2, with the range being 1–6 and the mean at 3.5. Mean total body surface area involved, from the burn injury, 16.1%. In terms of mechanism, the most common type of burn was flame (51%), followed by scald (25%), contact (8%), electrical (6%), and chemical (4%). Mean time to treat after injury was 45.7 months, with a median of 15.8 months. Length of followup was 4.4 months after the last session.

 Regarding treatment, we primarily utilized the following platforms: PDL (*n* = 305 patients, mean area treated: 68.6 cm^2^), CO_2_ (*n* = 122, 98.8 cm^2^), and IPL (*n* = 12, 35 cm^2^). Four patients underwent removal of persistent hair follicles with the Alexandrite laser, *n* = 2, and the light-sheer diode laser, *n* = 2. Thirty-three sessions out of 382, or 9%, included intralesional steroid injection for refractory hypertrophic scars or keloids. Adverse events were observed in 17 patients and included such major complications as laryngospasm (1), arrhythmia (1), blistering (2), and cellulitis (1), as well as such minor complications as hypopigmentation (8), hyperpigmentation (3), and postprocedure oral herpetic infection (1). Univariate analysis with chi-square and Student's *t *test revealed that increased Fitzpatrick skin type was associated with pigmentation complications. Pulsed-dye laser therapy in scald patients was associated with blister formation, and CO_2_ laser therapy was associated, not surprisingly, with postoperative pain.

 Changes in the burn scar were measured by the Vancouver Scar Sale (VSS), which is a validated instrument that objectively quantifies abnormalities in pigmentation, hyperemia, thickness, and pliability, producing scores that range from 0 (best) to 15 (worst) [22]. To capture the patient's subjective perception of the scar, we also developed a functional scar score, the UNC Scar Sale, that measures pruritis, dysesthesias, paresthesias, and stiffness, with a range of 0 (best) to 12 (worst). Both the PI and the second author, who were blinded to previous scores but not previous therapies, collected data prospectively. Treatment protocols were determined based upon the clinical needs of the patients.

 Laser treatments significantly improved both the objective components and subjective perception of hypertrophic burn scars. VSS decreased from 11.3 to 7.1 after one session, dropping further to 5.5 after the last session (both *P* < 0.001). Best-fit linear and logarithmic modeling indicates that the VSS would drop from 11.3 to 9.0 over the course of the study period, if the procedures were not performed and time was the only variable to consider. The UNC Scar Scale decreased from 6.0 to 3.4 after one session, with the final score of 2.2 after the last session (both *P* < 0.001). Best-fit linear and logarithmic modeling indicates that the UNC Scar Scale would decrease from 6.0 to 4.8 over the course of the study period, if the procedures were not performed and time was the only variable to consider. These data strongly support the conclusion that laser therapies can dramatically and quickly improve the functional components of hypertrophic burn scars. Determining the optimal timing and type of therapy will require more robust trials, but both the signs and symptoms of hypertrophic burn scars, as measured by objective and subjective instruments, significantly improved after treatment.

## 5. Case Studies

**Patient 1**

**Figure 1:**
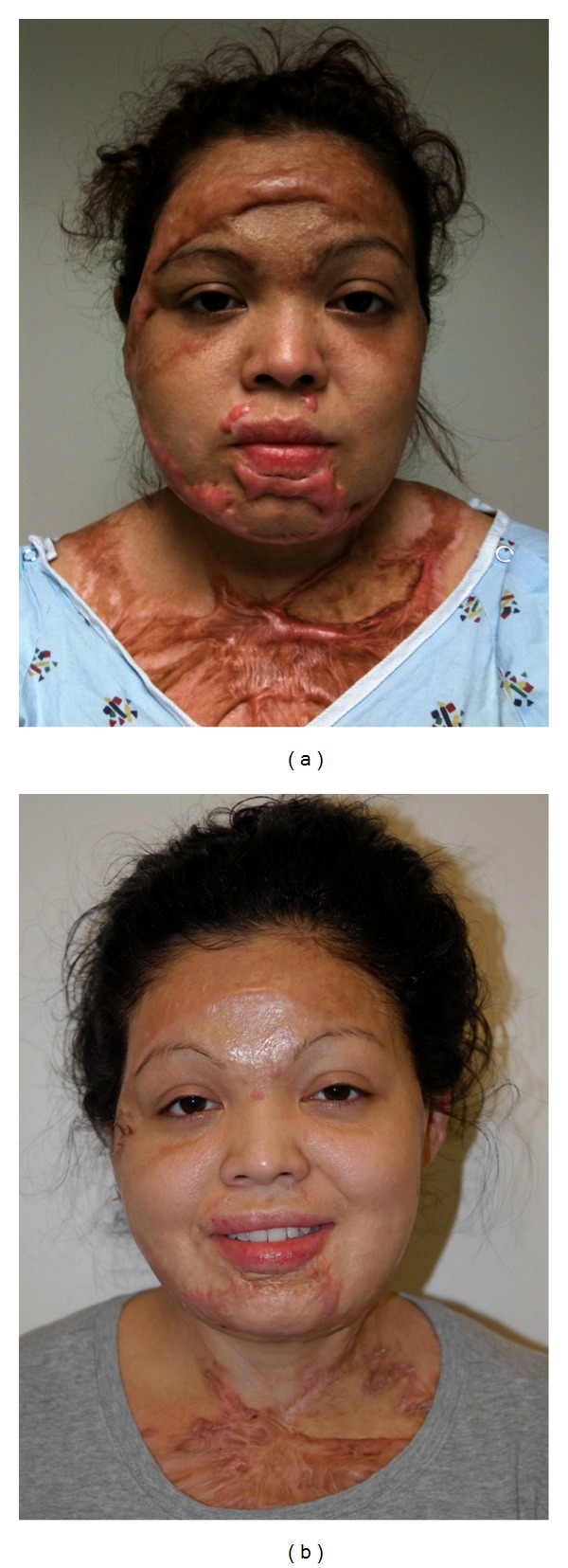


This 17-year-old sustained a 50% TBSA thermal burn, after her clothing caught on fire from a propane grill. Her reconstruction included tissue rearrangement, PDL × 5, CO_2_ laser × 2, and multiple steroid injections. She is now a freshman enrolled at the University of North Carolina and would like to pursue a career in journalism (Figure 1).

**Patient 2**

**Figure 2:**
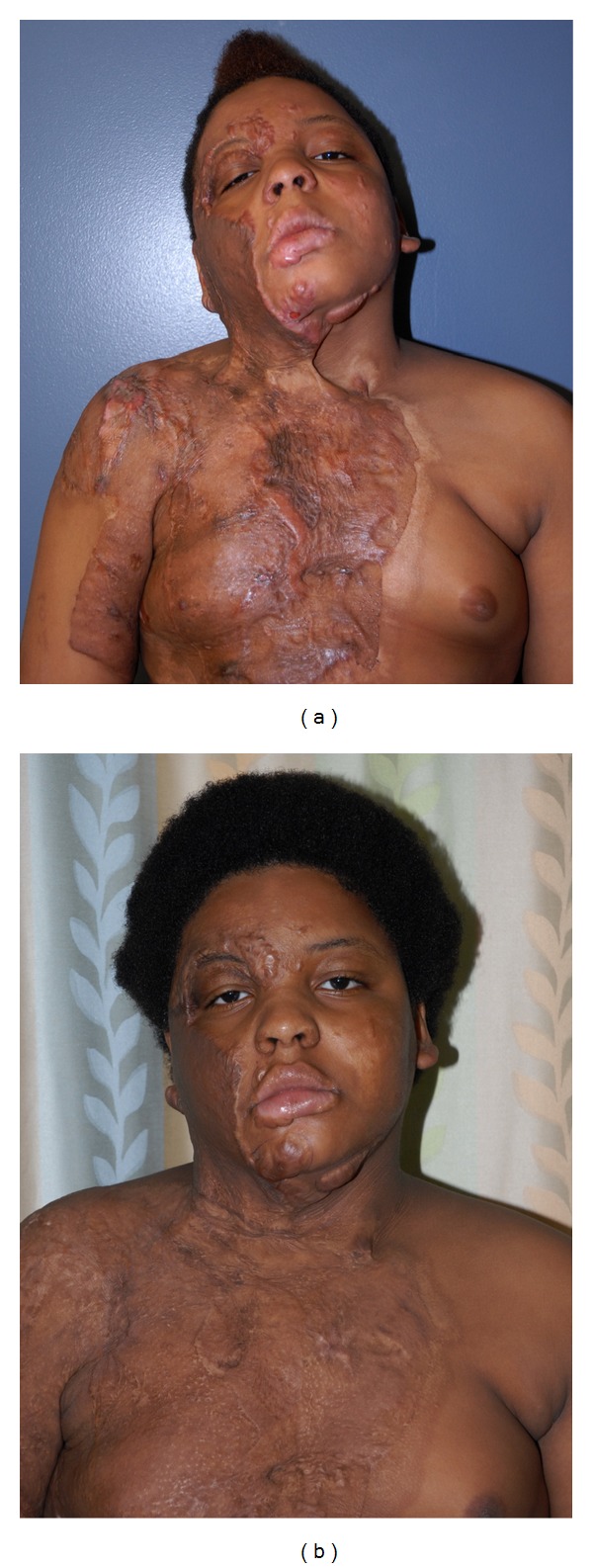


This 12-year-old boy sustained a 23% TBSA thermal burn, after his shirt caught on fire, when he was playing with matches. His reconstruction has included PDL × 3, CO_2_ laser × 1, and multiple steroid injections. He is now a candidate for surgical release of the neck contracture (Figure 2).

**Patient 3**

**Figure 3:**
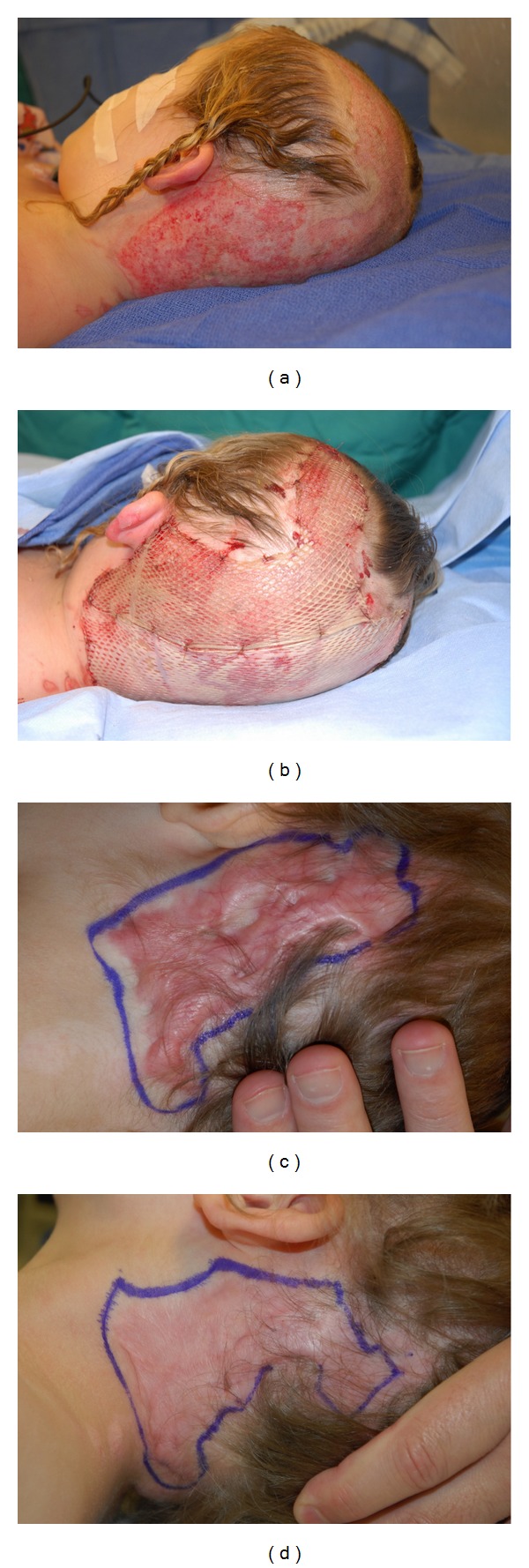


This 5-year-old girl sustained a 4% TBSA grease burn to her scalp and neck. After coverage with meshed xenograft, the patient developed severe hypertrophic scar formation that was successfully treated with PDL × 4 and no steroid injections (Figure 3).

**Patient 4**

**Figure 4:**
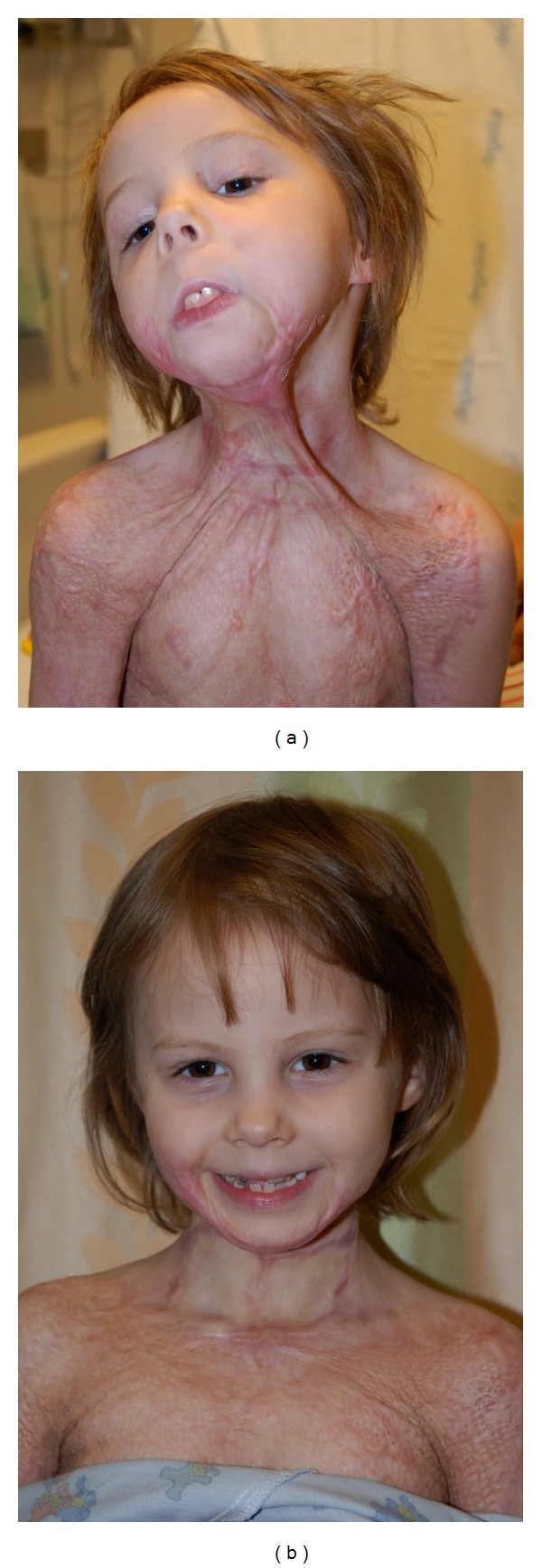


This 6-year-old girl sustained a 35% TBSA scald injury and subsequently developed a severe neck contracture. She underwent treatment with PDL × 3 prior to release of her neck and coverage with a full-thickness skin graft. Use of the PDL prevented the need to use tissue expanders or even a free flap, to correct this deformity (Figure 4).

**Patient 5**

This 25-year-old construction worker sustained a 10% TBSA burn from an electrical flash while laying shingles on a roof. He subsequently developed severe hypertrophic scars of both hands, intrinsic tightness, and no ability to abduct his digits. He underwent PDL × 2 and CO_2_ × 3 to both hands, with small full-thickness skin grafts to line his 2nd, 3rd, and 4th web spaces. He now has almost full range of motion with normal strength and has returned to work. His only restrictions are the need to wear protective gloves and no lifting greater than 50 pounds (Figure 5).

## 6. Aesthetic Considerations

 Although laser therapies can improve the functional problems of hypertrophic burn scars, no treatment algorithms have emerged to reliably treat burn scar dyschromias, which represent an unsolved cosmetic concern for many burn patients. Intense pulsed light (IPL) has been used successfully to treat other hyperpigmentation disorders, such as melasma and solar elastosis, but this modality has not been studied extensively in the treatment of burn scar dyschromias. Given the potential for improvement with IPL, we sought to determine whether or not this technology would help to correct these pigmentation abnormalities. We investigated both the clinical efficacy of and patient satisfaction with IPL in the treatment of burn scar dyschromias [23].

 Patients with symptomatic burn scar dyschromia were selected for treatment with IPL and excluded from this study if areas of hyperpigmentation had been treated previously with topical lightening agents, such as hydroquinone, tretinoin, or triamcinolone. With fluences ranging from 10–22 joules/cm^2^ and filters ranging from 560–640 nm, we used the LUME 1 platform (Lumenis) to target pigmented lesions in the middermis. Patients were pretreated with topical lidocaine (4–12%) only if they experienced discomfort. Procedures were performed in our out-patient clinic. No prophylactic antibiotics were given. Patients were not charged for the procedure. Providers assessed subjective improvement in dyschromia, and patients were queried for satisfaction, subjective efficacy, and willingness to pay.

 During 2011, 20 patients (11 females, 9 males; mean age 35.3 years, median Fitzpatrick skin type 4) underwent 23 IPL sessions in our clinic, 3.2 years after burn injury (flame 9, scald 5, contact 3, friction 1, ultrasound 1, electrical 1). Mean TBSA was 27.6%; mean treatment area was 90.1 cm^2^. Mean fluence was 15.4 J/cm^2^; the most common filter used was 590 nm. Adverse events were observed in 4 patients with significant pain and 2 patients with blistering. 16 patients had mild to moderate improvement in color, as rated by the providers. 4 patients had no response. Regarding patient perception, efficacy was rated at 4.5 (using a Likert scale, where 1 = significant worsening, 2 = slight worsening, 3 = no effect, 4 = mild improvement, and 5 = significant improvement). Patient satisfaction was rated at 4.4, with all patients recommending this to others and 87% of patients willing to have a repeat procedure. Patients reported that they would pay a mean of $7429 to remove their burn scars and valued each session to be worth a mean of $350 and a median of $1200. Mean length of followup after each procedure was 2.2 months.

 Intense pulsed light was helpful in the treatment of burn scar dyschromia, with a high level of patient satisfaction, but also created the potential for morbidity. Future prospective, validated trials will be necessary to generate guidelines for patient selection and treatment parameters. In the meantime, we offer IPL as a therapeutic option for treating burn scar hyperpigmentation, often in combination with topical therapy that includes hydroquinone, tretinoin, and fluocinolone (Tri-luma Cream, Galderma, Ft. Worth, TX, USA). Future efforts to address aesthetic concerns may also involve the use of very superficial, fractional CO_2_ resurfacing to help blend step-offs in color and texture. Until more data have been published, however, IPL should serve only as an alternative to other more established therapies, such as chemical lightening agents.

## 7. Logistical and Financial Considerations

 From the clinician's perspective, starting a laser practice, dedicated to the treatment of burn patients with hypertrophic scar formation, can be quite daunting. These patients may have significant medical comorbidities and must have a comprehensive medical work-up, as some medical conditions, such as diabetes, nicotine dependency, history of deep venous thrombosis, and neuropathic pain, may impact healing and recovery from laser treatments. On the other hand, these patients are largely grateful for even small, incremental improvements in their burn scars, especially if such symptoms as pruritis and dysesthesia can be alleviated. Nevertheless, managing expectations and providing a realistic perspective on final outcomes are critical to optimizing both patient and provider satisfaction.

 Almost all of the patients require treatment in the operating suite because of the following reasons: (1) patients may have difficult airways from previous tracheostomies or limited cervical extension due to their burn scars, (2) treatments are quite painful and many patients have pre-existing difficulties with chronic pain and posttraumatic stress disorder, (3) large areas approaching several hundred cm^2^ may need to be treated, which exceeds the ability to safely pretreat scars with topical anesthetics, (4) a large percentage of our patients are children, who do not have the cognitive maturity to remain still during a procedure, (5) treatments in the head and neck, where patients would be at risk for ocular complications from an errant laser pulse, also mandate a level of sedation that cannot be provided in the clinic, and (6) the surgeon can focus on providing the treatment, while the anesthesiologist or anesthetist can focus on providing anesthesia.

 Before the first patient is treated, the clinician must obtain access to the laser. In addition to taking a laser safety course and working closely with the institution's laser safety officer, the clinician must obtain institutional privileges to perform these procedures. Furthermore, the provider must develop intellectual competency in the physics, indications, treatment parameters, complications, safety protocols, and perioperative management of laser therapy. Acquiring the laser platforms may be done through rental, lease, or purchase, but the capital requirement can be considerable, as the value of the equipment, with the extended warranties, can easily approach $500,000 US. We recommend initially renting the equipment and transitioning to a lease, when demand for the procedures can be determined. Leasing also provides the opportunity to upgrade equipment as new technologies are developed and added to the platform. A final consideration that must be addressed, even before the first session, is who can operate the laser. This is often determined by institutional guidelines and state regulations. At our center, any midlevel provider or upperlevel provider can treat patients, but a physician must be in the operating suite at all times during the procedure. Furthermore, two individuals are required, at our ambulatory surgery center, to actually perform the procedure: the operator, who actually discharges the laser and aims the pulse at the targeted tissue, and the technician, who manages the settings of the laser and is immediately available to assist with any emergency situations, such as OR fire, device malfunction, or inadvertent discharge of the laser.

 The ability to collect revenue from these procedures is absolutely essential for the sustainability of the practice. Insurance companies legitimately do not cover most laser treatments for asymptomatic scars, especially when patients present with cosmetic concerns. However, we have found that almost all of the third-party payers do reimburse for laser treatment of burn scars, when functional problems such as contracture, stiffness, pruritis, and dysesthesia can be documented. We seek preauthorization and submit the results of our clinical consultation, combined with photographic documentation, for review. Direct, personal communication with medical directors at the insurance companies has facilitated this process; we have found that these individuals are quite helpful and interested in helping this group of patients. Because specific CPT codes for the laser treatment of burn scars do not exist, we use 17106, 17107, and 17108 (laser destruction of cutaneous vascular proliferative lesion, <10 cm^2^, 10–50 cm^2^, and >10 cm^2^), which serve as a proxy for what we do. The rationale for using these codes is that burn scars are hypervascular, hypertrophic, and hyperpigmented, all of which are due to a proliferative, neovascular, and hyperplastic response of the injured tissues. While not hemangiomas or vascular malformations, burn scars act like these lesions, due to similar pathophysiologic mechanisms, resulting in similar endpoints. Also important for documentation is using the correct ICD-9 diagnosis terminology, to signify that these scars are due to burn injury: 701.4, keloid or hypertrophic scar; 709.2, fibrosis of skin; 906.5–906.9, late effects of burn.

 One element that is particularly attractive to all stakeholders, patients, providers, and third-party payers, is that laser therapy for hypertrophic burn scars has the potential to dramatically reduce the cost of care. The surgical approach to management of burn scars can range from relatively simple laser treatments to very complex free flap reconstructions, depending upon the degree of contracture, pliability of the wound, and presence of such mitigating factors as ulceration and folliculitis. Allowable professional fees, for North Carolina Medicaid, range from $361 for a laser session to $2493 for the free flap reconstruction, the latter of which would consume 4–6 hours of OR time, 1-2 days of intensive care, and several additional days of step-down care before hospital discharge. The laser cases take one hour, including anesthesia induction/emergence, surgical procedure, transport time, and room turnover. Of course, patients with neck contractures may not be adequately treated with laser therapy alone. However, 1-2 laser sessions may preclude the need for any invasive surgery in patients with mild to moderate contractures and may permit less aggressive, and less costly, procedures, such as tissue rearrangement or skin graft, to be utilized in patients with moderate to severe contractures. For patients who suffer from severe pruritis, debilitating paresthesias, and chronic pain, laser treatment of hypertrophic burn scars almost always decreases pharmacologic requirements and allows some patients to discontinue many or all of their medications, such as narcotics, anxiolytics, antihistamines, and antidepressants. Patients who can wean off of these complex regimens faster require less clinic followup and are more successful with rehabilitative efforts and return to work faster.

## 8. Future Investigation

 While much knowledge about the effect of lasers on burn scars has been acquired over the past few years, this work is just beginning. Anecdotal experience, retrospective series, prospective before-after studies, and now our systematic review all support the use of laser and light-based therapies for the treatment of burn scars. However, prospective, blinded, randomized, controlled trials will be necessary for two reasons. First, because most burn scars tend to improve over time, albeit slowly, it will be necessary to tease out the variable of time from our interventions and focus on the effects of the therapies only. While laser treatments appear to reverse the hypertrophic response and provide substantial relief from pruritis and dysesthesias, long-term outcomes are still unknown. Does laser therapy reset our target endpoints, or do laser treatments just accelerate maturation of the burn scar and reduce the time needed for healing? Either way, patients will experience gains, through improved quality of life, earlier return to work, and more rapid wean from pharmacologic regimens that often include antipruritics, anxiolytics, antidepressants, and narcotics.

 The second driving force behind the need for robust RCTs is that clinicians need to develop best practices, which must take into account type of treatment and timing of therapies. For example, does early intervention improve outcome? If so, how early? Identifying those burn patients who are at risk for hypertrophic scars will allow clinicians to begin therapy earlier. Within a type of therapy, clinicians can vary several different parameters, such as fluence, pulse time, and density. The potential exists to treat different components of the burn scar, with different settings. Furthermore, different types of therapies, such as PDL and IPL, need to be compared with each other, as these technologies utilize different mechanisms of action and may work synergistically when used together.

## 9. Summary

In conclusion, several traditional and emerging technologies may significantly change how clinicians manage burn scars. Laser- and light-based therapies can be combined with each other to safely yield superior results, often at lower cost, by reducing the need for reconstructive surgery. Modulating the burn scar, through noninvasive and minimally invasive modalities, may replace conventional methods of burn scar excision. Such a paradigm shift in burn scar treatment may yield outcomes not previously possible or conceivable.

## Figures and Tables

**Figure 5:**